# Enhancing Scene Text Recognition with Encoder–Decoder Interactive Model

**DOI:** 10.3390/s25247684

**Published:** 2025-12-18

**Authors:** Yongbin Mu, Mieradilijiang Maimaiti, Miaomiao Xu, Wenkai Li, Wushour Silamu

**Affiliations:** 1 School of Computer Science and Technology, Xinjiang University, Urumqi 830017, China; muyongbin@stu.xju.edu.cn (Y.M.); miradeljan51@xju.edu.cn (M.M.); liwenkai@stu.xju.edu.cn (W.L.); 2 Xinjiang Laboratory of Multi-Language Information Technology, Xinjiang University, Urumqi 830017, China; 3 Xinjiang Multilingual Information Technology Research Centre, Xinjiang University, Urumqi 830017, China; 4 Joint International Research Laboratory of Silk Road Multilingual Cognitive Computing, Xinjiang University, Urumqi 830017, China

**Keywords:** scene text recognition, multi-scale dilated fusion attention, sequential encoder-decoder context fusion, encoder-decoder interactive model

## Abstract

Scene text recognition has significant application value in autonomous driving, smart retail, and assistive devices. However, due to challenges such as multi-scale variations, distortions, and complex backgrounds, existing methods such as CRNN, ViT, and PARSeq, while showing good performance, still have room for improvement in feature extraction and semantic modeling capabilities. To address these issues, this paper proposes a novel scene text recognition model named the Encoder–Decoder Interactive Model (EDIM). Based on an encoder–decoder framework, EDIM introduces a Multi-scale Dilated Fusion Attention (MSFA) module in the encoder to enhance multi-scale feature representation. In the decoder, a Sequential Encoder–Decoder Context Fusion (SeqEDCF) mechanism is designed to enable efficient semantic interaction between the encoder and decoder. The effectiveness of the proposed method is validated on six regular and irregular benchmark test sets, as well as various subsets of the Union14M-L dataset. Experimental results demonstrate that EDIM outperforms state-of-the-art (SOTA) methods across multiple metrics, achieving significant performance gains, especially in recognizing irregular and distorted text.

## 1. Introduction

Reading text in natural scenes is a key capability for building intelligent automated systems, as it provides rich and precise semantic information. With the rapid development of computer vision, the task of detecting and recognizing text in complex backgrounds—namely, scene text recognition (STR)—has gained widespread attention. STR has significant practical value in applications such as autonomous driving, augmented reality, retail analytics, education, and accessibility. Compared to traditional optical character recognition (OCR) [1], STR faces more diverse and severe challenges, including font and layout variations, irregular text shapes, complex backgrounds, lighting changes, and blur or distortion of images. Thus, despite its challenges, STR is a vital task and an important direction in advancing artificial intelligence.

**Figure 1:**
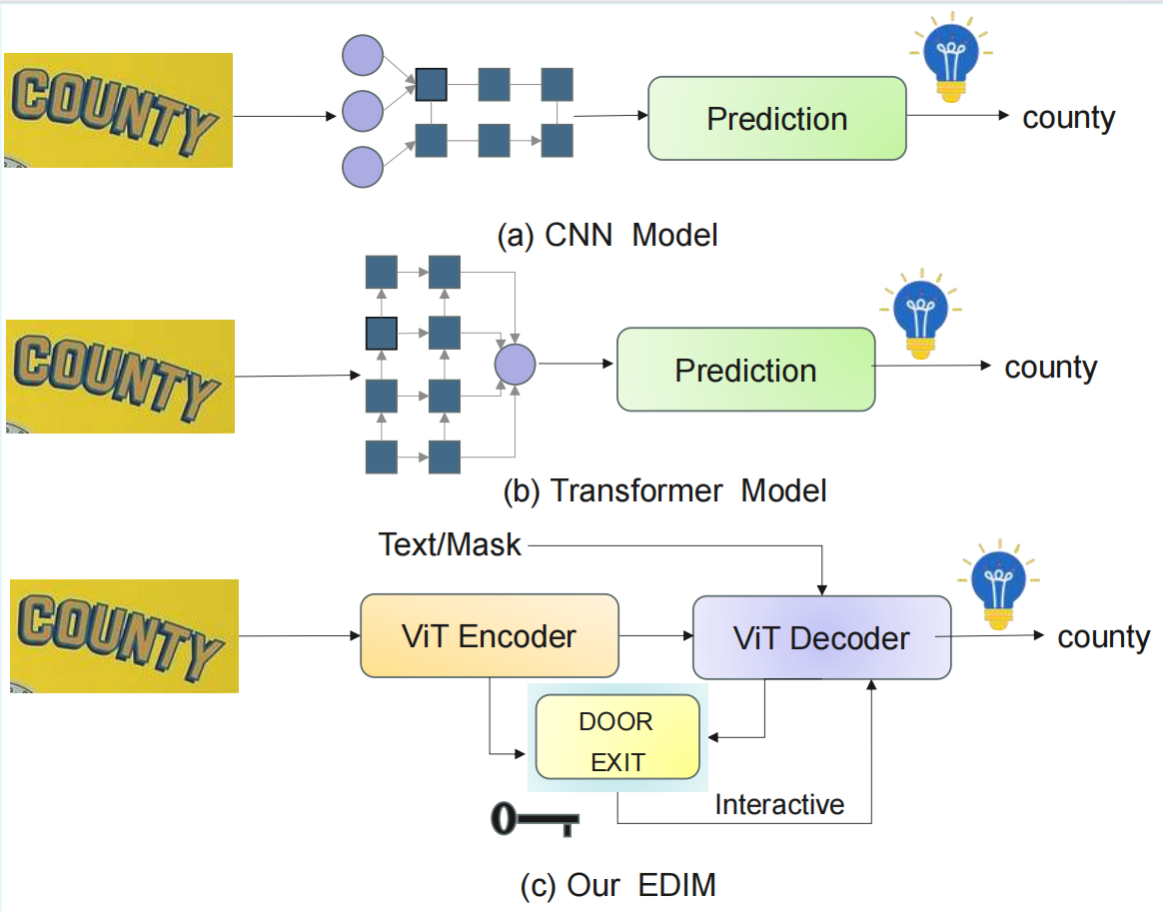
The evolution of scene text recognition methods: from the initial (**a**) CNN-based models, to the encoder–decoder architecture with Transformer (**b**) vision–language models, and finally to the novel encoder–decoder framework proposed in this work, (**c**) our EDIM.

To address these issues, researchers have proposed many innovative methods. Early works used N-gram models with convolutional neural networks (CNNs) to enhance character feature extraction. Although these improved accuracy, they struggled to model character context. With deep learning advances, attention-based methods emerged, combining CNN [2] features with attention mechanisms (e.g., RARE [3], SEED [4]) as shown in Figure 1a, to improve the dependencies of the model. However, their performance on large-scale datasets remained limited. The introduction of the Connectionist Temporal Classification (CTC [2]) framework, such as in CRNN [2], improved alignment and recognition. Later, encoder–decoder architectures became mainstream, with models such as PARSeq [5] and MGP [6] using Vision Transformer (ViT [7]) encoders and Transformer decoders, as shown in Figure 1b. However, they often do not use language information. More recent methods, such as CLIP4STR [8] and DTROCR [9], integrated large pre-trained language models to improve semantic understanding, boosting performance but increasing model size and computation.

This paper proposes a novel STR model, EDIM, shown in Figure 1c, to address limitations in multi-scale feature extraction, distortion handling, and semantic modeling. The encoder combines MSFA and Transformer layers to enhance features across scales, while the decoder introduces a SeqEDCF mechanism for efficient encoder–decoder semantic interaction, leading to more accurate recognition. The main contributions of this paper are summarized as follows:

1. To address the limitation of insufficient multi-scale feature extraction in existing encoders for complex scenes, we propose a Multi-scale Dilated Fusion Attention (MSFA) module. This module systematically integrates features from varying receptive fields and employs a channel–spatial attention mechanism, significantly enhancing the model’s robustness against scale variations, distortions, and complex backgrounds.
2. To overcome the static and inefficient semantic interaction prevalent in current encoder–decoder architectures, we design a Sequential Encoder–Decoder Context Fusion (SeqEDCF) mechanism. This innovation facilitates dynamic, gated global semantic interaction, thereby improving the modeling of long-range dependencies and contextual consistency.
3. Building upon these components, we construct the EDIM. Extensive experiments demonstrate that our model performs better on multiple standard benchmark datasets, particularly for irregular text recognition, while maintaining a favorable balance between model complexity and inference speed.

The remainder of this paper is organized as follows: Section 2 reviews related work on scene text recognition. Section 3 details the proposed EDIM methodology, including the MSFA module and SeqEDCF mechanism. Section 4 presents experimental results, comparisons with state-of-the-art methods, and ablation studies. Finally, Section 5 concludes the paper and discusses future work.

## 2. Related Work

With the continuous development of deep learning technology, most STR methods have become based on deep neural networks and generally adopt strategies that integrate semantic information to improve recognition performance. Depending on whether semantic information is incorporated, existing STR methods can be roughly categorized into two types: context-independent methods [2,5,7] and context-aware methods [2,5,8,10,11,12,13].

Context-independent STR methods mainly focus on extracting visual features from images and directly predicting characters without incorporating semantic or contextual information. The output characters are conditionally independent of one another, resulting in weaker robustness when dealing with occlusion, incomplete characters, or degraded scenes. Early methods often relied on traditional techniques such as SIFT and HOG, or used convolutional neural networks (CNNs) for feature extraction with CTC [2] decoding, as exemplified by Rosetta. With the rise of the Transformer architecture in vision tasks, models such as Vision Transformer (ViT [7]) have been introduced into STR, leading to a series of context-independent methods based on pure encoders, such as ViTSTR, SVTR [14], and MGP [6]-STR. ViTSTR achieves efficient decoding based on a pure ViT encoder, and SVTR [14] integrates multi-scale features to enhance encoder representation, while MGP-STR further improves recognition through an adaptive addressing module. These methods offer structural simplicity and inference efficiency, but due to a lack of global semantic and contextual modeling, they underperform in semantically rich or complex scenarios. As a result, researchers have begun introducing language modeling and semantic information to enhance robustness and generalization in complex scenes.

Context-aware STR methods significantly improve recognition in complex scenarios such as distorted, occluded, or blurred text by fusing visual features with language semantics. Early methods often used CNNs [2] to extract features. They combined them with BiLSTM and attention for sequence modeling and decoding (e.g., TRBA [10]), but suffered from limited inference efficiency due to the sequential nature of RNNs. With the rise of Transformer, researchers proposed methods to enhance internal language modeling: some added independent language branches to the encoder (e.g., CDistNet [15]) for joint training of visual and semantic cues; others, like VisionLAN, adopted masked language modeling during training to selectively mask characters and enhance contextual understanding. PARSeq [5] introduced a permutation language model (PLM), leveraging dual-stream attention in a single Transformer decoder for bidirectional context modeling and iterative refinement without extra semantic branches. Similarly, ABINet [16] improves predictions through staged training of visual–semantic dual branches and iterative correction in decoding. Methods like TrOCR [11], LevOCR [12], CLIP4STR [8], and CLIP-OCR [8] use large-scale pre-trained vision–language models (e.g., CLIP [8], BERT [13]) to inject language knowledge into visual features, forming a predict-and-refine pipeline that boosts semantic reasoning. Although these approaches significantly improve accuracy and robustness, they generally rely on extensive annotated data and high computational costs.

Second, the encoder–decoder interaction is often one-time or static (e.g., merely passing a final context vector), failing to fully leverage the multi-scale visual features generated throughout the encoding process. Our work is designed to directly address these challenges by focusing on developing a more efficient encoder–decoder interaction architecture that enables deeper, more dynamic visual–semantic fusion without significantly increasing computational burden.

## 3. Method

**Figure 2:**
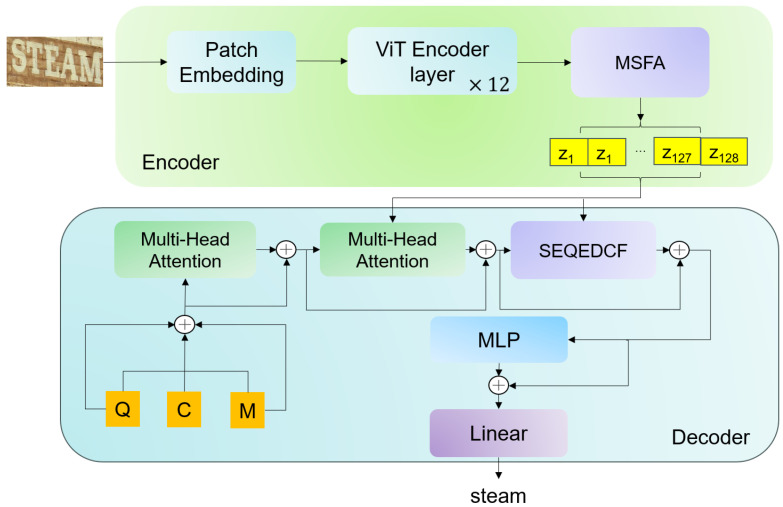
Architecture overview of EDIM. The encoder first processes the input image through a Vision Transformer (ViT), which includes patch embedding and Transformer blocks, followed by the Multi-Scale Fusion Attention (MSFA) module. The decoder uses the proposed SeqEDCF mechanism to fuse memory (M, from encoder), query (Q, predicted tokens), and context (C, hidden state) for autoregressive generation.

This paper proposes a novel scene text recognition (STR) model, named the Encoder–Decoder Interactive Model (EDIM), based on a robust encoder–decoder architecture. The encoder first converts the input image into a token sequence via a convolutional patch embedding layer, where each token represents a local image region. It then applies twelve Transformer encoder blocks, each combining multi-head self-attention and feed-forward networks to capture complex patterns. A Multi-Scale Fusion Attention (MSFA) module is integrated into the encoder to enhance multi-scale feature representation. For the decoder, we adopt an architecture similar to PARSeq [5], but introduce a novel sequence encoder–decoder gated fusion mechanism (SeqEDCF). The overall framework is illustrated in Figure 2.

### 3.1. Encoder

**Figure 3:**
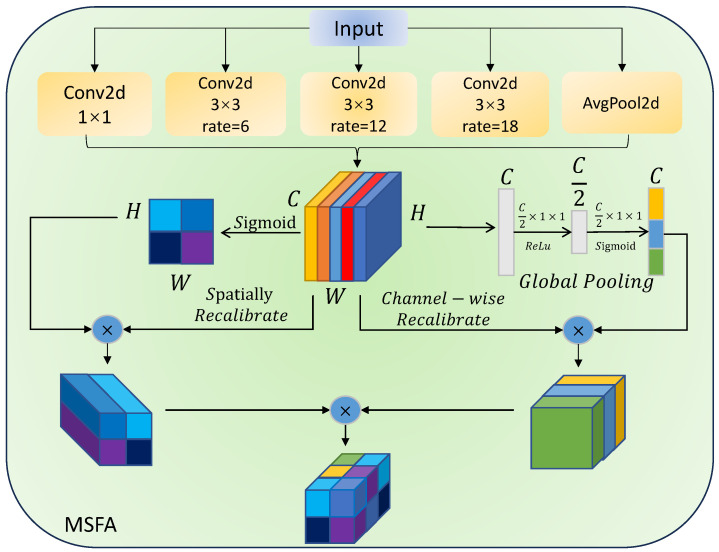
MSFA (Multi-Scale Fusion Attention) extracts multi-scale features using convolutions with varying dilation rates, specifically set to {1, 6, 12, 18}, along with global pooling, thereby achieving the fusion of local and global information. Subsequently, channel and spatial attention mechanisms are applied to weight the features, emphasizing key regions while suppressing noise. This enhances the model’s ability to perceive targets of varying scales and shapes in complex visual tasks, such as scene text recognition (STR).

The encoder of EDIM primarily consists of two components: a standard Vision Transformer (ViT) [7] that performs patch embedding and self-attention modeling, and the proposed Multi-Scale Fusion Attention (MSFA) module that enhances multi-scale contextual understanding. Specifically, the ViT is composed of 12 sequential Transformer encoder layers, each containing a multi-head self-attention mechanism and a feed-forward neural network, with layer normalization applied before each sub-layer (pre-norm configuration) to stabilize training and maintain feature distribution consistency. The MSFA module operates on the output features of the ViT to further enrich their representational capacity by jointly modeling multi-scale spatial contexts, global dependencies, and channel–spatial interactions, as illustrated in Figure 3.

The selection of dilation rates [1,6,12,18] is informed by the statistical characteristics of text instances in standard STR datasets. In typical input images resized to 32×128, character heights occupy approximately 50–88% of the vertical dimension, while horizontal spans vary significantly due to font style and word length. To effectively capture both local stroke patterns and global contextual layout, we adopt a geometric progression in dilation rates that provides a balanced coverage of spatial scales: small (d=1) for fine details, medium (d=6) for single-character context, and large (d=12,18) for multi-character or curved text modeling. This configuration empirically achieves a favorable trade-off between representational capacity and model complexity.

The input image $X\in R^{H\times W\times C}$ is first processed by the patch embedding module (16×16), where it is divided into fixed-size image patches and mapped into a high-dimensional feature space to obtain a sequence of patch features:

(1) $$Z_{\mathrm{patch}\;}=\mathrm{Patch}\mathrm{Embed}\left(X\right)\in R^{N\times D},$$

where N denotes the number of patches and D represents the embedding dimension. This patch feature sequence is then fed into the Vision Transformer (ViT [7]) backbone, where multiple layers of self-attention mechanisms capture global dependencies. Each layer comprises a multi-head self-attention (MHA) module and a feed-forward network (MLP).

This patch feature sequence is then fed into successive Transformer encoder layers. The inputs to the first multi-head self-attention (MHA) layer include the query vector q, contextual information c, and a mask m. Unlike the left-to-right decoding order in Transformer, the mask m used during training is generated by a permuted language model, primarily to introduce varied decoding orders during the decoding phase. Let $Z^{\left(l\right)}$ denote the input to the *l*-th Transformer encoder layer, with $Z^{\left(0\right)}=Z_{\mathrm{patch}}$. The computation in each layer can be formulated as

(2) $$Z^{\prime (l)}=Z^{\left(l\right)}+\mathrm{MHA}\left(\mathrm{LayerNorm}\left(Z^{\left(l\right)}\right)\right),$$

(3) $$Z^{\left(l+1\right)}=Z^{\prime (l)}+\mathrm{MLP}(\mathrm{LayerNorm}(Z^{\prime (l)})).$$

After proceeding through all *L* layers, the final output is represented as $Z_{\mathrm{ViT}}=Z^{\left(L\right)}\in R^{N\times D}$, which serves as the global feature sequence.

On this basis, to further enhance the multi-scale contextual modeling capability, a Multi-Scale Multi-Branch Fusion Attention (MSFA) module is introduced. MSFA contains multiple convolutional branches with different dilation rates:

(4) $$F_{i}={\mathrm{Conv}}_{3\times 3}^{d=d_{i}}\left(Z_{ViT}\right),$$

where i=1,…K, to extract features under different receptive fields, while a global pooling branch is used to capture overall contextual information:

(5) $$F_{g}=\;\mathrm{Interpolate}\;\left(\mathrm{GlobalAvgPool}\;\left(Z_{\mathrm{ViT}}\right)\right).$$

Finally, the features from all branches are concatenated along the channel dimension and fused through a 1 × 1 convolution, forming the final multi-scale enhanced encoded features:

(6) $$Z_{\mathrm{enc}}={\mathrm{Conv}}_{1\times 1}\left(\mathrm{Concat}\left(F_{1},F_{2},\ldots ,F_{K},F_{g}\right)\right),$$

where $Z_{\mathrm{enc}}$ represents the enhanced feature map that will be passed to the decoder.

### 3.2. Decoder

In this model, the decoder is designed to efficiently utilize the multi-scale enhanced features output by the encoder to generate the target character sequence. The decoder adopts a decoding method similar to that of PARSeq [5]. During the training phase, PARSeq’s permutation language modeling approach generates different masking orders, referred to as the M part in the model. This method allows the decoder to adapt to different decoding orders during decoding, thereby better understanding contextual information. However, the decoding order follows the standard left-to-right sequence during testing and inference.

**Figure 4:**
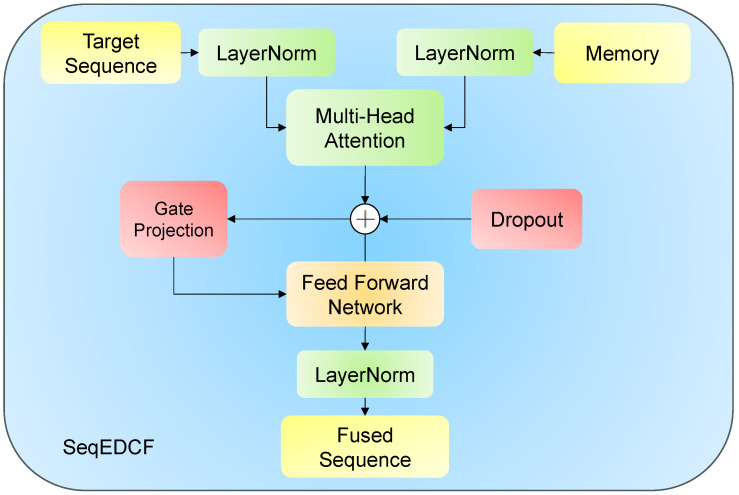
This figure illustrates the structure of the SeqEDCF mechanism: the target sequence and memory features first pass through layer normalization, and then undergo feature interaction via multi-head attention. Next, they go through residual connection, gated projection, feed-forward network, and another layer normalization step. Finally, the fused sequence features are output to enhance the decoding performance.

The decoder consists of two multi-head attention (MHA) modules, the SeqEDCF module, and a multilayer perceptron (MLP). The SeqEDCF module employs eight attention heads with a model dimension of $d_{\mathrm{model}}=512$ and a feed-forward network hidden dimension of 2048. The Hebing module comprises a 1×1 convolution followed by a ReLU activation and a sigmoid gating mechanism. The overall architecture of the SeqEDCF module is illustrated in Figure 4.

The calculation expression of the first multi-head attention is

(7) $$h_{1}=Q+\mathrm{MHA}\left(Q,C,M\right)\in R^{\left(T+1\right)\times d_{\mathrm{model}}},$$

where *Q*, *C*, and *M* represent the query vectors (used to retrieve the most relevant contextual information for the current decoding step), the contextual information (enhanced features $Z_{enc}$ from the encoder output), and the mask generated by the permutation language modeling, respectively. T is the length corresponding to *C*.

Next, the second multi-head attention module further models the dependencies within the target sequence itself. Its inputs are the previous step’s output $h_{1}$ and the final multi-scale enhanced encoded features $Z_{enc}$ obtained from the encoder. Its expression is as follows:

(8) $$h_{2}=h_{1}+\mathrm{MHA}\left(h_{1},Z_{\mathrm{enc}},Z_{\mathrm{enc}}\right)\in R^{\left(T+1\right)\times d_{\mathrm{model}}}.$$

Subsequently, before being fed into SeqEDCF, the output $h_{2}$ of the second multi-head attention and the final encoded features $Z_{enc}$ from the encoder are each subjected to layer normalization (LayerNorm) by the decoder. Their expressions are as follows:

(9) $$h_{2}^{\prime }=\mathrm{LayerNorm}\left(h_{2}\right),$$

(10) $$Z_{\mathrm{enc}}^{\prime }=\mathrm{LayerNorm}\left(Z_{\mathrm{enc}}\right).$$

This step helps stabilize the training process and improve numerical stability.

Next, a multi-head cross-attention mechanism establishes the dependency between the target position and image features. This module uses $h_{2}^{\prime }$ as the query, utilizes $Z_{enc}$ as both the key and value, and combines residual connection and dropout to obtain the cross-attention output. The expression is

(11) $$h_{3}=h_{2}+\mathrm{Dropout}\left(\mathrm{MHA}\left(h_{2}^{\prime },Z_{\mathrm{enc}}^{\prime },Z_{\mathrm{enc}}^{\prime }\right)\right).$$

After obtaining the preliminary fused features $h_{3}$, the process enters the SeqEDCF module. This module includes a gated projection mechanism to introduce adaptive modulation information for the target sequence. Its expression is

(12) $$G=\sigma (W_{g}h_{2}),$$

where $W_{g}\in R^{D\times D}$ is a learnable matrix, and σ(·) denotes the sigmoid activation function used to generate the gating weights.

Next, the gated output is added to the fused features, followed by further modeling through a feed-forward network; finally, layer normalization is applied to obtain the fused output of SeqEDCF. The expression is

(13) $$h_{4}=\mathrm{LayerNorm}\left(\mathrm{FFN}(G+h_{3})\right).$$

Finally, the result is fed into an MLP and a linear layer to obtain the final character prediction. The expression is:

(14) $$\mathrm{logit}=\mathrm{Linear}\left(\mathrm{MLP}\left(h_{4}\right)\right).$$

From the decoder’s decoding process, it can be seen that the SeqEDCF module effectively associates encoder features with decoder features, achieving global information interaction between the encoder and decoder and effectively leveraging global semantic information.

### 3.3. Comparison with Proposed Method

To better position our work within the existing literature, we provide explicit comparisons between our proposed methods and related approaches.

#### 3.3.1. MSFA vs. Existing Multi-Scale Methods

Traditional multi-scale approaches in computer vision, such as those in Inception networks [17] and ASPP [18], primarily focus on expanding receptive fields through parallel convolutional branches with different kernel sizes or dilation rates. While effective for general object recognition, these methods are not specifically optimized for the unique challenges of STR, where text instances exhibit extreme scale variations within the same image.

Our MSFA module advances beyond these approaches in three key aspects:

- Task-specific dilation selection: We carefully select dilation rates [1, 6, 12, 18] based on empirical analysis of text scale distributions in standard STR benchmarks, unlike fixed or arbitrary selections in general-purpose methods.
- Integrated attention mechanism: We incorporate channel and spatial attention to dynamically recalibrate feature importance, addressing the background clutter and noise issues prevalent in natural scene text.
- Global context integration: We explicitly include a global average pooling branch to capture image-level context, which is particularly important for resolving ambiguities in challenging text instances.

#### 3.3.2. SeqEDCF vs. Conventional Cross-Attention Mechanisms

Cross-attention has become a standard component in encoder–decoder architectures for sequence-to-sequence tasks. However, most existing STR methods employ straightforward cross-attention implementations where the decoder queries the encoder’s features in a static interaction.

Our SeqEDCF mechanism introduces several innovations:

- Dynamic gated fusion: The incorporation of a learnable gating mechanism ($G=\sigma (W_{g}h_{2})$) allows adaptive modulation of feature fusion throughout the decoding process, allowing context-sensitive integration at each step.
- Multi-stage interaction: Unlike single-pass attention mechanisms, SeqEDCF facilitates ongoing dialogue between encoder and decoder features through its sequential application in the decoding process.
- Stabilized training: The explicit layer normalization and residual connections in SeqEDCF ensure stable training while enabling deep feature interaction.

#### 3.3.3. Architecture-Level Innovation

While many recent STR methods focus on enhancing visual features or improving language modeling, our EDIM represents a holistic approach that systematically addresses both aspects through the synergistic combination of MSFA and SeqEDCF. This integrated design philosophy distinguishes our work from methods that excel in one area but underperform in others, such as ViTSTR (strong visual features but limited semantic modeling) or ABINet (sophisticated language modeling but complex architecture).

## 4. Experiments

This section outlines the experimental setup, including datasets, preprocessing, training/evaluation protocols, and metrics. We then present results and compare the proposed method with state-of-the-art (SOTA) approaches using these metrics and computational cost indicators. Ablation studies are conducted to assess the impact of training permutations on test accuracy. Finally, case studies illustrate the model’s specific performance.

### 4.1. Datasets

In this paper, we evaluate our EDIM method on multiple benchmark test sets, which include the following: 1. Six commonly used regular and irregular test sets: ICDAR 2013 (IC13 [19]), Street View Text (SVT [20]), IIIT5K-Words (IIIT5K [21]), ICDAR 2015 ([22]), Street View Text-Perspective (SVTP [23]), and CUTE80 [24]. IC13 and IC15 each have two versions—one containing IC13 857 and IC15 1811, and another containing IC13 1015 and IC15 2077. We chose the former versions for this work. 2. The latest Union14M-L [25] benchmark (U14M) test set, which contains seven challenging subsets: Curved Text (Curve), Multi-Oriented Text (MO), Artistic Text (Artistic), Background-Free Text (Cless), Salient Text (Salient), Multi-Word Text (MW), and General Text (General).

During the training phase, we trained our model on real-world datasets due to the increase in real-world scene datasets, enabling the model to exhibit stronger recognition capability. We mainly trained it on two large-scale real-world training sets: the Real dataset [5] and the Union14M-Filterr [26] dataset.

### 4.2. Experimental Setup

To comprehensively evaluate model performance under diverse and potentially imbalanced data distributions, we employ a multi-faceted assessment framework encompassing both recognition quality and computational efficiency metrics.

#### 4.2.1. Evaluation Metrics

Three key metrics are adopted to provide complementary insights into model performance:

- Accuracy: The primary metric measuring sequence-level exact match, where a prediction is considered correct only if the entire output string perfectly matches the ground truth:
  (15) $$\mathrm{Accuracy}=\frac{\mathrm{Number}\;\mathrm{of}\;\mathrm{Correct}\;\mathrm{Predictions}}{\mathrm{Total}\;\mathrm{Number}\;\mathrm{of}\;\mathrm{Samples}}\times 100\%$$
- 1 - Normalized Edit Distance (1 - NED): A character-level metric that quantifies the similarity between predicted and ground truth sequences, providing finer-grained assessment when perfect sequence matching is not achieved. This metric is particularly valuable for evaluating performance on challenging or imbalanced data subsets:
  (16) $$1-\mathrm{NED}=1-\frac{\mathrm{Edit}\;\mathrm{Distance}(P,G)}{\max(|P|,|G|)}$$
  where *P* denotes the predicted sequence and *G* represents the ground truth.
- Confidence Score: The average prediction confidence across all samples, reflecting the model’s calibration quality and reliability in real-world deployment scenarios.

#### 4.2.2. Computational Efficiency

To assess practical applicability, we additionally report

- Model Size (Parameters): Total trainable parameters in millions (M);
- Inference Speed (FPS): Frames processed per second on NVIDIA V100 GPU.

#### 4.2.3. Training Configuration

The model is trained using the AdamW optimizer with OneCycleLR learning rate scheduling for the first 14 epochs, featuring linear warm-up followed by cosine annealing. Stochastic Weight Averaging (SWA) is employed for the remaining 6 epochs to enhance generalization. All experiments use a batch size of 384 across 2 NVIDIA Tesla V100 GPUs (Xinjiang Laboratory of Multi-Language Information Technology, Xinjiang University, Urumqi, China) for 20 epochs, with Python 3.10.16 and PyTorch 2.7.0.

This comprehensive evaluation protocol ensures rigorous assessment of both recognition capability and practical utility across varied application scenarios.

### 4.3. Comparative Analysis with Existing Methods

Since the training sets in this paper include the Real training [5] set and the Union14M-Filter [26] training set, we conduct training on both datasets separately and then evaluate the relevant metrics. The results for these two training sets are presented in distinct tables to ensure clarity:

Training on the Real training set: Table 1 compares our method with state-of-the-art approaches on six standard English benchmark test sets.

Training on the Union14M-Filter training set: Table 2 compares the six standard test sets and seven challenging subsets from the Union14M-L benchmark.

This separate presentation allows for a comprehensive evaluation under different training data conditions and ensures transparency in our experimental design.

First, we train our model on the Real training set and compare it with state-of-the-art methods on English datasets, such as PARSeq [5] and MGP [6], as well as some classic methods on English datasets like ViTSTR [7], ABINet [16], and SRN [27]. The results are shown in Table 1.

Table 1 summarizes the experimental results on several widely used English benchmark datasets: IIIT5K, SVT, IC13, IC15, SVTP, and CUTE80. Since our model is trained on the Real training set, we compare it only with methods that are also trained exclusively on the same dataset. As shown in Table 1, our method, EDIM, achieves strong performance across all six standard benchmarks, particularly on the two irregular text datasets—SVTP and CUTE80. This indicates that our MSFA module significantly enhances the encoder’s feature extraction capability, enabling superior performance on challenging, non-horizontal text instances. Moreover, EDIM achieves competitive accuracy with relatively low computational cost, as evidenced by its favorable FLOPs compared to other state-of-the-art methods.

Furthermore, we conduct additional experiments using Union14M-Filter [26] from SVTRv2 [26] as the second training set, a real-world dataset derived from Union14M-L. Due to potential data overlap between the Real set and Union14M-L, which may lead to data leakage and compromise the fairness of comparison, we adopt the filtered version—Union14M-Filter—to ensure a more rigorous evaluation. The corresponding results are presented in Table 2.

**Table 2:** Models trained on the Union14M-Filter training set, tested on six commonly used regular and irregular test sets and seven challenging test subsets, and compared with other existing methods.

Method	Venue	Common Benchmarks						Union14M Benchmarks								Size/FPS	
		IIIT5K	SVT	IC13	IC15	SVTP	CUTE80	Curve	Multi-O	Artistic	Context	Salient	Multi-W	General	Avg	Size (M)	FPS
ASTER [28]	TPAMI19	96.1	93.0	94.9	86.1	87.9	92.0	70.9	82.2	56.7	62.9	73.9	58.5	76.3	68.77	19.0	67.1
MORAN [29]	PR19	96.7	91.7	94.6	84.6	85.7	90.3	51.2	15.5	51.3	61.2	43.2	64.1	69.3	50.83	17.4	59.5
AutoSTR [30]	ECCV20	96.8	92.4	95.7	86.6	88.2	93.4	72.1	81.7	56.7	64.8	75.4	64.0	75.9	70.09	6.0	82.6
RoScanner [31]	ECCV20	98.5	95.8	97.7	88.2	90.1	97.6	79.4	68.1	70.5	79.6	71.6	82.5	80.8	76.07	48.0	64.1
ABINet [16]	CVPR21	98.5	98.1	97.7	90.1	94.1	96.5	80.4	69.0	71.7	74.7	77.6	76.8	79.8	75.71	36.9	73.0
PARSeq [5]	ECCV22	98.9	98.1	98.4	90.1	94.3	98.6	87.6	88.8	76.5	83.4	84.4	84.8	84.3	84.26	23.8	52.6
MATRN [32]	ECCV22	98.8	98.3	97.9	90.3	95.2	97.2	82.2	73.0	73.4	76.9	79.4	77.4	81.0	77.61	44.3	46.9
MGP-STR [6]	ECCV22	97.9	97.8	97.1	89.6	95.2	96.9	85.2	83.7	72.6	75.1	79.8	71.1	83.1	78.65	148	120
CPPD [33]	Preprint	99.0	97.8	98.2	90.4	94.0	99.0	88.8	78.7	76.5	92.8	85.3	81.9	83.5	81.93	27.0	125
LPV [34]	IJCAI23	98.6	97.8	98.1	89.8	93.7	97.6	86.2	78.7	75.8	80.2	82.9	81.6	82.9	81.18	30.5	82.6
MAERec [25]	ICCV23	99.2	97.8	98.2	90.4	94.3	98.3	89.1	87.1	79.0	84.2	86.3	85.9	84.6	85.17	35.7	17.1
LISTER [35]	ICCV23	98.8	97.5	98.6	90.0	94.4	96.9	78.7	68.8	73.7	81.6	74.8	82.4	83.5	77.64	51.1	44.6
CDistNet [15]	IJCV24	98.7	97.1	97.8	89.6	94.0	95.9	81.7	71.2	72.6	78.2	79.9	79.7	81.1	77.77	43.3	15.9
BUSNet [36]	AAAI24	98.3	98.1	97.8	90.2	95.3	96.5	83.0	82.3	70.8	77.9	78.8	71.2	82.6	78.09	32.1	83.3
OTE [37]	CVPR24	98.6	96.6	98.0	90.1	94.0	97.2	86.0	75.8	74.6	74.7	81.0	65.3	82.3	77.10	20.3	55.2
SVTRv2 [26]	ICCV25	99.2	98.0	98.7	91.1	93.5	99.0	90.6	89.0	79.3	86.1	86.2	86.7	85,1	86.14	19.8	143
**EDIM (Ours)**	–	**99.0**	**99.0**	**98.6**	**89.7**	**96.6**	**97.9**	**89.4**	**91.6**	**78.7**	**85.5**	**86.2**	**86.3**	**85.9**	**86.23**	**23.8**	**14.8**

Observing the data in Table 2, we see that our method continues to demonstrate strong performance. On the six commonly used regular and irregular test sets, SVTP [23] surpasses existing methods by an average of approximately two percentage points, while on SVT [20], it exceeds others by about one point on average. On IIIT5K [21], IC13 [19], IC15 [22], and CUTE80 [24], our results are comparable to other state-of-the-art approaches. Furthermore, our method also performs well on the seven challenging test subsets. On the Multi-Oriented Text (MO) subset, it outperforms other methods by about one percentage point on average, while achieving broadly comparable results to other strong approaches across the remaining six challenging subsets. Compared with the latest SVTRv2 (2025), our approach achieves higher accuracy—1% on SVT [20], 3% on SVTP [23], and 2.6% on the Multi-Oriented Text subset—while maintaining comparable results on the other test datasets. In addition, our model contains fewer parameters, providing better efficiency and deployment advantages.

To enhance the interpretability of the SeqEDCF mechanism, we conduct a qualitative analysis of its adaptive behavior under various challenging scenarios. In cases of low-resolution or heavily occluded text, the gating mechanism consistently assigns high weights to contextual information from the decoder state (gate values > 0.7), leveraging linguistic context to resolve visual ambiguities. In contrast, for clear and well-aligned text, the mechanism shifts focus toward visual features from the encoder (gate values < 0.3), ensuring precise character-level localization. In curved text recognition, SeqEDCF maintains consistent attention along the text trajectory throughout the decoding process, effectively modeling spatial progression along nonlinear paths. For semantically ambiguous cases—such as visually similar characters (‘c’ and ‘e’) under poor illumination—the mechanism achieves balanced gate values (approximately 0.4–0.6), allowing complementary cues from both modalities to jointly contribute to the final decision. These observations demonstrate that SeqEDCF facilitates a dynamic and context-sensitive dialogue between the encoder and decoder throughout the decoding process.

#### Comprehensive Metric Analysis for Imbalanced Data

To address the challenge of evaluating model performance under imbalanced data distributions, we conduct a detailed analysis using complementary metrics beyond overall accuracy. This analysis provides deeper insights into the model’s behavior across diverse data characteristics and subset sizes. We employed the Union14M-L dataset for training.

Analysis on Highly Imbalanced Union14M-L Dataset: As shown in Table 3, the Union14M-L dataset exhibits significant class imbalance, with the General subset containing 387,287 samples while other challenging subsets range from only 779 to 2426 samples. Despite this imbalance, EDIM demonstrates remarkable consistency across all metrics:

- The Artistic subset (898 samples), while achieving 78.73% accuracy, maintains a high 1 - NED of 93.95%, indicating that most recognition errors are minor character-level deviations rather than complete failures.
- On the Multi-Oriented subset (1369 samples), EDIM achieves excellent performance across all metrics (accuracy: 91.60%, 1 - NED: 97.22%, confidence: 92.19%), demonstrating robust handling of oriented text despite limited training data.
- The Multi-Word subset shows the highest 1 - NED (98.02%), suggesting exceptional character-level accuracy in recognizing longer text sequences.
- Confidence scores remain stable across all subsets (83.26–92.19%), indicating consistent model calibration regardless of subset size or difficulty level.

Analysis on Standard Benchmarks: Table 4 presents results on balanced benchmark datasets. The close alignment between accuracy (96.22%) and 1 - NED (98.76%) demonstrates that EDIM’s predictions are character-wise accurate even when not perfectly matching the ground truth. The high confidence scores (95.77%) further validate the model’s well-calibrated uncertainty estimation.

Cross-Dataset Consistency: The complementary metrics reveal EDIM’s consistent performance patterns across both imbalanced and balanced datasets. The minimal gaps between accuracy and 1 - NED scores across all evaluations confirm that the model’s advantages are genuine and not artifacts of specific data distributions. This comprehensive analysis substantiates EDIM’s robustness and reliability for real-world deployment scenarios involving naturally imbalanced data.

### 4.4. Analysis of Computational Efficiency

Beyond recognition accuracy, we also evaluated the computational efficiency of our EDIM. As summarized in Table 2, our model contains 25.8 million parameters and achieves an inference speed of 14.8 FPS. EDIM strikes a favorable balance between performance and efficiency compared to other state-of-the-art methods. For instance, while achieving superior or comparable accuracy on irregular text benchmarks (SVTP [23] and CUTE80 [24]), our model is significantly more efficient than methods that rely on extensive pre-trained vision–language models (e.g., CLIP4STR [8], TrOCR [11]), which often have parameter counts an order of magnitude higher. The lightweight design of our core components—the MSFA module for efficient multi-scale feature extraction and the SeqEDCF mechanism for dynamic feature fusion without recurrent connections—contributes to this manageable computational burden. This makes EDIM accurate and more practical for real-world deployment scenarios with limited computational resources.

To directly address the efficiency in processing a batch of images, we estimate the total processing time for 100 images. Our EDIM, with an inference speed of 14.8 FPS, would require approximately 6.76 s (100 images/14.8 FPS ≈ 6.76 s). This demonstrates a highly competitive throughput. In contrast, methods with lower FPS (e.g., around 10 FPS) would require about 10 s for the same task. In comparison, those with higher FPS might trade off recognition accuracy, especially on challenging irregular text datasets, as shown in Table 2. The performance of our model on such a batch would be fast and accurate, maintaining the high recognition rates reported in Table 1 and Table 2 across both regular and irregular text. This balance is crucial for practical applications where speed and accuracy are paramount.

The comprehensive metrics presented in Table 3 and Table 4 provide additional validation of EDIM’s robustness. The strong performance across all three metrics–particularly the high 1 - NED scores–confirms that our model’s advantages extend beyond simple sequence matching to include superior character-level recognition capability. This is especially evident in challenging scenarios like curved and multi-oriented text, where EDIM maintains high 1 - NED scores despite the inherent difficulties of these categories.

Furthermore, the well-calibrated confidence scores across all subsets indicate that EDIM provides reliable uncertainty estimation, making it suitable for real-world applications where understanding prediction certainty is crucial for downstream decision-making.

### 4.5. Comprehensive Ablation Studies

To thoroughly validate the effectiveness of our proposed EDIM framework, we conducted extensive ablation studies focusing on three key aspects: (1) the individual and combined contributions of MSFA and SeqEDCF modules, (2) analysis of different dilation rate configurations in MSFA, and (3) direct comparison between SeqEDCF and standard attention mechanisms.

#### 4.5.1. Component-Wise Analysis

The component-wise analysis reveals several key insights in Table 5:

- Individual Contributions: MSFA and SeqEDCF significantly improve performance when used independently. MSFA contributes primarily through enhanced multi-scale feature representation, while SeqEDCF improves semantic modeling and contextual consistency.
- Synergistic Effect: The combination of MSFA and SeqEDCF yields the highest performance gains (+0.30%), demonstrating that the enhanced visual features from MSFA are particularly well-utilized by the sophisticated fusion mechanism in SeqEDCF.
- Challenging Scenarios: The performance improvements are more pronounced on the seven challenging datasets, indicating that our modules are particularly effective for complex text recognition scenarios involving irregular shapes, distortions, and complex backgrounds.

#### 4.5.2. MSFA Dilation Rate Analysis

The performance advantage of the MSFA module can be attributed to the statistical characteristics of text scale distribution: its multi-scale design directly addresses the extreme scale variations of text instances in natural scenes. Meanwhile, the improvement of the SeqEDCF mechanism stems from its dynamic gating fusion capability, which enables the model to adaptively adjust the contribution weights of visual and semantic information based on input complexity—a behavior validated through our qualitative analysis in Table 6.

Note: Dilation Set A: [1, 2, 3, 4]; Set B: [1, 3, 5, 7]; Set C: [1, 6, 12, 18]; Set D: [6, 12, 18, 24]; Set E: [1, 12, 24, 36].

The dilation rate analysis provides crucial insights into multi-scale feature design:

- Small Rates (Set A): Effective for local detail preservation but insufficient for capturing broader contextual information, particularly limiting performance on datasets with significant scale variations.
- Moderate Rates (Set C): Our selected configuration achieves optimal balance, with progressive scaling from character-level (rate 1) to word-level (rate 6) and scene-level (rates 12–18) context capture.
- Large Rates (Set E): Capture extensive contextual information but sacrifice local detail precision, particularly affecting performance on small or distorted text instances.

The chosen configuration [1, 6, 12, 18] explicitly addresses the multi-scale nature of scene text, where individual characters, words, and text blocks coexist within the same image while maintaining computational efficiency.

#### 4.5.3. SeqEDCF vs. Standard Attention Mechanisms

The attention mechanism comparison reveals distinct advantages of our SeqEDCF approach in Table 7:

- **Standard Cross-Attention**: It provides basic encoder–decoder interaction but lacks adaptive capabilities, particularly struggling with irregular text (SVTP: 92.3%, CUTE80: 96.1%).
- **Enhanced Variants**: Multi-head and residual variants offer incremental improvements but maintain the fundamental limitation of static context transfer.
- **SeqEDCF Advantages**: The gating mechanism enables dynamic, context-aware feature fusion throughout decoding, achieving substantial improvements on challenging datasets (SVTP: +3.6%, CUTE80: +2.0% over standard cross-attention).

#### 4.5.4. Computational Efficiency and Practical Considerations

The computational analysis demonstrates that our EDIM framework achieves an excellent balance between performance and efficiency in Table 8:

- **Reasonable Overhead**: The complete EDIM introduces approximately 20% additional parameters while maintaining competitive inference speed (14.8 FPS).
- **Performance-9Efficiency Trade-off**: The 2.21% accuracy gain justifies the computational cost, particularly for applications requiring high recognition accuracy on challenging text.
- **Practical Viability**: The maintained FPS rate ensures real-time applicability in practical deployment scenarios such as autonomous driving and document digitization.

In summary, our comprehensive ablation studies validate both proposed modules’ effectiveness and synergistic combination. The MSFA module significantly enhances multi-scale feature representation through optimized dilation rate selection, while SeqEDCF provides sophisticated dynamic fusion capabilities that substantially outperform standard attention mechanisms. The combined EDIM framework achieves state-of-the-art performance while maintaining practical computational efficiency.

### 4.6. Case Study

By conducting recognition experiments on irregular, handwritten, curved, occluded, distorted, and perspective text images across four models (CRNN [2], ViT [7], CDistNet [15], and our method), we evaluated the accuracy differences between our model and other approaches, as demonstrated in Table 9. Table 9 reveals that while the conventional CRNN method struggles to recognize text in complex scenarios accurately, ViT significantly improves recognition performance through its superior encoder–decoder architecture. Our model achieves outstanding accuracy comparable to state-of-the-art CDistNet [15] when processing irregularly shaped, handwritten, curved, occluded, and distorted images. This indicates that our MSFA (Multi-Scale Feature Aggregation) module effectively assists the model in extracting corresponding features from images, enabling precise recognition of such challenging text. Furthermore, the SEQEDCF (Semantic-Enhanced Query-Based Dynamic Context Fusion) module associates global semantic information, allowing the model to fully leverage semantic cues for more accurate recognition of occluded images.

### 4.7. Cross-Lingual Applicability Analysis

To evaluate the generalization capability of EDIM in multi-lingual scenarios, we conducted text recognition experiments on Uyghur-language images in addition to English. The study employed a self-constructed Uyghur dataset collected from various real-world environments in Urumqi, Xinjiang, China. Outdoor scenarios included bus stops, road signs, storefront signage, banners, product packaging, and hospital boards, while indoor environments encompassed printed notices, exhibition labels, and shopping mall directories. The dataset was rigorously partitioned into training and validation sets at an 8:2 ratio, with the validation set simultaneously serving as the test set.

To address data scarcity limitations, we implemented extensive augmentation techniques—including geometric transformations, imaging perturbations, color adjustments, and grid distortion—generating 1.76 million synthetic images, of which 1.31 million were retained for training. This comprehensive dataset authentically captures characteristic challenges such as typographic diversity, character ligatures, complex backgrounds, and low-resolution conditions, thereby establishing a valuable benchmark for low-resource scene text recognition.

Furthermore, to accommodate the distinctive character set of the Uyghur script, we replaced the original 94-character English vocabulary (comprising 0–9, a–z, A–Z, and 32 special characters) with 32 Uyghur alphabetic letters and appended an additional special token <end>. The input image size was standardized to 32 × 128 pixels, with a maximum sequence length set to 25 tokens. All experiments were conducted on a single Tesla V100 GPU, utilizing mixed-precision training through PyTorch Lightning. The model was optimized using AdamW with a OneCycleLR learning rate scheduler.

As demonstrated in Table 10, our model achieves competitive performance in the Uyghur comparative study, with an accuracy margin of approximately one percentage point compared to the most recent baseline models. When evaluated against 2024 benchmarks, our method shows improvements of 2–3 percentage points, while significantly outperforming earlier approaches by considerable margins. These results substantiate that our approach maintains consistent effectiveness not only for English text recognition but also for the morphologically distinct Uyghur script, demonstrating its cross-lingual applicability. This robust performance across linguistically diverse writing systems suggests strong potential for future extension to other languages.

## 5. Conclusions

This paper proposes a novel STR model, EDIM, to address challenges in natural STR, such as multi-scale variation, complex deformation, and insufficient semantic modeling capability. The model is designed based on an encoder–decoder architecture. The encoder integrates MSFA, effectively enhancing the model’s ability to extract features from different scales and shapes, improving perception and robustness against complex backgrounds, distortions, and curved texts. The design choices in this paper are not only empirically validated, but also grounded in a statistical analysis of text scale distribution in STR datasets, with enhanced theoretical interpretability provided through qualitative analysis of the SeqEDCF mechanism. The dilation rate configuration in MSFA systematically covers the hierarchical range of text scales—from local stroke patterns to global layout—while the dynamic gating mechanism in SeqEDCF enables adaptive feature fusion based on the complexity of the input.

The main benefits of our method are threefold: (1) It achieves superior recognition accuracy, especially on challenging irregular text benchmarks, outperforming existing state-of-the-art methods while maintaining favorable computational efficiency; (2) the proposed MSFA and SeqEDCF modules provide an effective solution for multi-scale feature extraction and dynamic encoder–decoder interaction without significantly increasing computational burden; (3) our model demonstrates excellent practicality with balanced performance in terms of accuracy, parameter count, and inference speed.

However, our approach still has some limitations. First, while our method maintains a good balance between accuracy and efficiency, there remains a trade-off between computational cost and performance gains, particularly when dealing with extremely distorted or low-resolution text images. Second, the current architecture does not explicitly incorporate text rectification modules, which might limit its performance on severely perspective-distorted texts. Third, the method has primarily been validated on English datasets, and its effectiveness on other languages with different character sets requires further investigation. Additionally, while our model excels in handling multi-scale and distorted text under normal-resolution conditions, its performance may degrade on extremely low-resolution or heavily compressed images. This is because the patch-based ViT encoder and the dilated convolutions in the MSFA module rely on sufficient spatial detail to extract discriminative features. In such cases, character strokes may become indistinguishable after patch embedding, leading to ambiguous representations that hinder accurate recognition. Future work could explore adaptive patch sizing or lightweight super-resolution modules to enhance robustness in low-quality scenarios.

For future work, we plan to address these limitations through several directions: (1) Developing more lightweight and efficient variants of the MSFA and SeqEDCF modules to optimize the speed–accuracy trade-off further; (2) integrating explicit geometric transformation modules to handle more severe text distortions; (3) extending our approach to multi-lingual scenarios, particularly for complex script systems like Uyghur, Arabic, and other minority languages; (4) exploring the application of our method to video text recognition by incorporating temporal modeling capabilities; (5) exploring integration with recent document-understanding Transformers like DiT [38] and Dount [39], which leverage large-scale self-supervised pre-training for richer layout and semantic modeling, potentially enhancing our approach in broader document analysis scenarios.

Experimental results demonstrate that EDIM performs excellently on multiple public benchmark datasets, significantly outperforming existing state-of-the-art methods, especially in recognizing irregular and distorted texts. Ablation studies verify the critical roles of the MSFA and SeqEDCF modules in improving model performance and their synergistic effect. Our work provides a valuable reference for the development of efficient and accurate STR systems in practical applications.

## Figures and Tables

**Table 1:** Models trained on the Real training set, tested on six commonly used regular and irregular test sets, and compared with other methods.

Method	Training Data	FLOPs (G)	Test Datasets # of Samples					
			IIIT5k	SVT	IC13	IC15	SVTP	CUTE80
CRNN [2]	Real	1.3	94.6	90.7	94.1	78.5	86.9	89.7
TRBA [10]	Real	2.1	96.4	91.6	96.6	81.6	90.2	92.7
ViTSTR [7]	Real	4.2	98.3	96.2	97.9	88.8	91.6	96.5
ABINet [16]	Real	3.8	98.6	97.8	98.0	90.2	93.9	97.7
PARSeq [5]	Real	2.4	99.1	97.9	98.3	90.7	95.7	98.3
MGP [6]	Real	3.7	98.4	98.3	98.6	91.1	96.6	97.9
EDIM (Ours)	Real	**2.5**	**99.33**	**98.9**	**98.5**	**91.4**	**96.7**	**98.3**

**Table 3:** Performance analysis on Union14M-L subsets, reporting accuracy, 1 - normalized edit distance (1 - NED), and model confidence (all in %).

Dataset	Samples	Accuracy	1 - NED	Confidence
Artistic	898	78.73	93.95	83.26
Contextless	779	85.49	97.08	89.03
Curved	2426	89.41	97.31	90.84
General	387,287	85.88	93.41	89.16
Multi-Oriented	1369	91.60	97.22	92.19
Multi-Word	824	86.29	98.02	88.72
Salient	1583	86.23	96.90	89.57
**Combined**	**395,166**	**85.91**	**93.48**	**89.16**

**Table 4:** Performance analysis on standard benchmark datasets, reporting accuracy, 1 - normalized edit distance (1 - NED), and model confidence (all in %).

Dataset	Samples	Accuracy	1 - NED	Confidence
IIIT5K	3000	99.03	99.72	97.20
SVT	647	99.07	99.68	96.27
IC13	1015	98.62	99.47	97.75
IC15	2077	89.74	96.58	92.52
SVTP	645	96.59	99.03	95.41
CUTE80	288	97.92	99.28	97.13
**Combined**	**7672**	**96.22**	**98.76**	**95.77**

**Table 5:** Component ablation study. ✓ indicates module usage, while ✗ indicates exclusion. Evaluation conducted on six common datasets (Real [5]) and seven challenging datasets (Union [26]).

Configuration		Average Accuracy (%)		Performance Gain (%)
MSFA	SeqEDCF	Six Common	Seven Challenging	
✗	✗	96.3	85.9	-
✓	✗	96.5	86.0	+0.15
✗	✓	96.7	86.1	+0.20
✓	✓	**96.9**	**86.2**	**+0.30**

**Table 6:** Analysis of different dilation rate configurations in the MSFA module.

Dilation Rates	IIIT5K	SVT	IC13	IC15	SVTP	CUTE80
Dilation Set A	98.7	97.8	98.1	90.1	95.2	97.1
Dilation Set B	98.9	98.2	98.3	90.5	95.8	97.6
**Dilation Set C (Ours)**	**99.0**	**98.9**	**98.5**	**91.4**	**96.7**	**98.3**
Dilation Set D	98.8	98.1	98.2	90.3	95.9	97.4
Dilation Set E	98.5	97.9	98.0	89.8	95.1	96.9

**Table 7:** Comparison of SeqEDCF with standard attention approaches.

Attention Mechanism	IIIT5K	SVT	IC13	IC15	SVTP	CUTE80
Standard Cross-Attention	98.2	96.8	97.9	88.5	92.3	96.1
Multi-Head Cross-Attention	98.4	97.2	98.1	89.0	93.1	96.5
Cross-Attention + Residual	98.6	97.5	98.2	89.3	93.8	96.9
**SeqEDCF (Ours)**	**99.2**	**98.3**	**98.5**	**90.5**	**95.9**	**98.1**

**Table 8:** Computational efficiency analysis of different configurations.

Configuration	Parameters (M)	FPS	Accuracy Gain (%)
Baseline (standard ViT)	21.5	18.2	-
+ MSFA only	24.1	16.8	+1.56
+ SeqEDCF only	23.8	15.9	+1.78
**Full EDIM**	**25.8**	**14.8**	**+2.21**

**Table 9:** Demo image analysis across different models: The sample images contain irregular, handwritten, curved, occluded, distorted, and perspective text. The parts marked in red indicate incorrect predictions; GT refers to the ground truth label.

Input		Prediction/Model			
GT	Image	CRNN [2]	ViT-S [7]	CDistNet [15]	EDIM (Ours)
neptuno	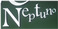	neptuno	neptuno	neptuno	neptuno
pniol	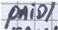	pniol	pniol	pniol	pniol
haircuts	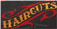	haircuts	haircuts	haircuts	haircuts
the	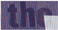	th0	the	the	the
3rdave	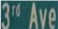	3ave	3rdave	3rdave	3rdave
arcakaise	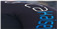	......	arcakaise	arcakaise	arcakaise
nsuranc	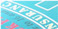	......	nsnranc	nsuranc	nsuranc
knives	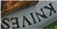	......	kniaes	knives	knives

**Table 10:** Comparison experiment with other methods on the self-built Uyghur dataset.

Method	Venue	Accuracy (%)	Parameter (M)
CRNN [2]	TPAMI16	92.20	16.2
ASTER [28]	TPAMI19	85.26	19.0
TRBA [10]	ICCV19	94.83	49.8
AutoSTR [30]	ECCV20	87.71	6.0
ViTSTR [7]	ICDAR21	91.81	21.7
PARSeq [5]	ECCV22	94.42	23.8
MGP-char [6]	ECCV22	94.66	85.9
CDistNet [15]	IJCV24	92.96	43.3
OTE [37]	CVPR24	91.58	20.3
VS-2DF	PRCV25	**95.93**	19.8
EDIM (Ours)	-	**94.91**	25.6

## Data Availability

Data are contained within the article.
